# Ultralight Iontronic Triboelectric Mechanoreceptor with High Specific Outputs for Epidermal Electronics

**DOI:** 10.1007/s40820-022-00834-4

**Published:** 2022-03-29

**Authors:** Hai Lu Wang, Zi Hao Guo, Xiong Pu, Zhong Lin Wang

**Affiliations:** 1 Beijing Institute of Nanoenergy and Nanosystems, Chinese Academy of Sciences, Beijing, 101400 People’s Republic of China; 2 School of Nanoscience and Technology, University of Chinese Academy of Sciences, Beijing, 100049 People’s Republic of China; 3 Center on Nanoenergy Research, School of Physical Science and Technology, Guangxi University, Nanning, 530004 People’s Republic of China; 4 CUSTech Institute of Technology, Wenzhou, 325024 Zhejiang People’s Republic of China; 5 School of Materials Science and Engineering, Georgia Institute of Technology, Atlanta, GA 30332 USA

**Keywords:** Ultralight, Iontronic, Triboelectric mechanoreceptor, Power density, Epidermal electronics

## Abstract

The pursuit to mimic skin exteroceptive ability has motivated the endeavors for epidermal artificial mechanoreceptors. Artificial mechanoreceptors are required to be highly sensitive to capture imperceptible skin deformations and preferably to be self-powered, breathable, lightweight and deformable to satisfy the prolonged wearing demands. It is still struggling to achieve these traits in single device, as it remains difficult to minimize device architecture without sacrificing the sensitivity or stability. In this article, we present an all-fiber iontronic triboelectric mechanoreceptor (ITM) to fully tackle these challenges, enabled by the high-output mechano-to-electrical energy conversion. The proposed ITM is ultralight, breathable and stretchable and is quite stable under various mechanical deformations. On the one hand, the ITM can achieve a superior instantaneous power density; on the other hand, the ITM shows excellent sensitivity serving as epidermal sensors. Precise health status monitoring is readily implemented by the ITM calibrating by detecting vital signals and physical activities of human bodies. The ITM can also realize acoustic-to-electrical conversion and distinguish voices from different people, and biometric application as a noise dosimeter is demonstrated. The ITM therefore is believed to open new sights in epidermal electronics and skin prosthesis fields.

**Supplementary Information:**

The online version contains supplementary material available at 10.1007/s40820-022-00834-4.

## Highlights

- An ultralight, ultrathin iontronic triboelectric mechanoreceptor (ITM) is proposed with high specific outputs for multi-functional epidermal applications.
- Precise vital signals and physical activities monitoring of human bodies are noninvasively implemented by the ITM.
- Acoustic-to-electrical energy conversion is realized by the ITM, and biometric applications such as voices differentiation and the noise dosimeter are demonstrated.

**Supplementary Information:**

The online version contains supplementary material available at 10.1007/s40820-022-00834-4.

## Graphical abstract

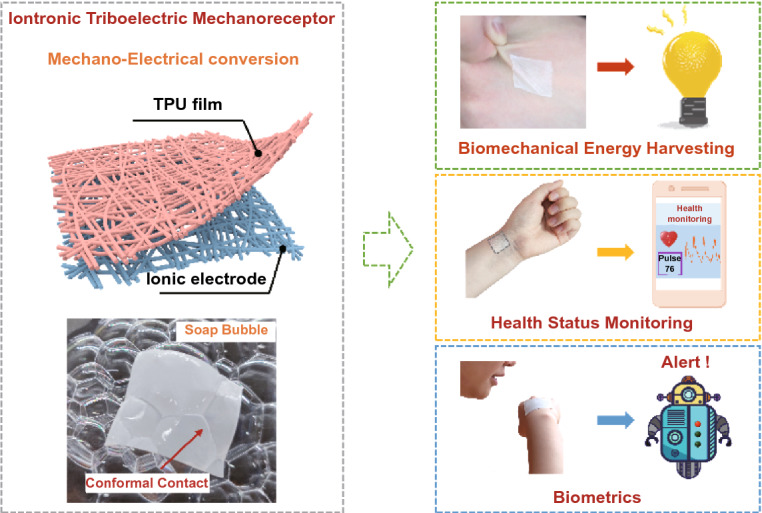

## Introduction

The human body perceives the world with the aid of sophisticated receptors responsive to different environmental stimuli, among which, the mechanoreceptors in human skin are crucial components for the somatosensory system. Mechanoreceptors, which transduce external mechanical stimuli into intracellular signals, enable our sensation of touch, press, stretching even acoustic vibrations [1–4]. Therefore, epidermal electronics that replicate tactile sensing features of human skin have attracted substantial interests because of their potential significances in humanoid robotics and prosthetics [5–7]. Diverse artificial epidermal mechanoreceptors have been developed by converting environmental stimuli into digital electric signals [8]. From the perspective of long-term applicability, several critical factors should be satisfied for epidermal electronics. Firstly, sufficient softness and skin conformability should be achieved to form intimate and conformal contact between epidermal devices and the skin, whereas ultra-conformal contact is not easy to implement since human skin comprises many non-flat surfaces and fine topology structures [9]. Secondly, reliable flexibility and stretchability are required to sustain large skin deformation and to avoid measurement artefacts arising from relative motion between the devices and the skin [10]. Thirdly, it is better for on-skin devices to be breathable in order to maintain the thermal-moisture regulation functions of the human skin and to minimize possible discomfort or irritation for long-term wearing by human beings [11]. Current artificial epidermal mechanoreceptors are still yet to achieve these features simultaneously, since it remains difficult to minimize the device architecture/configuration without sacrificing the sensitivity and stability.

Another major challenge for epidermal electronics is the power/energy predicament. Generally, epidermal mechanoreceptors rely heavily on external energy devices; nevertheless, it is hard for power suppliers to achieve stretchability or breathability compatible with the epidermal sensors [12, 13]. Therefore, self-powered triboelectric-based sensors have been emerging recently, since they generate electrical outputs by themselves in response to mechanical stimuli; while for mostly reported epidermal electronics, they still need external electricity sources [4, 14–18]. Furthermore, triboelectric sensors can be readily flexible by employing thin-film polymer or polymer-based composite materials [19, 20]. In particular, triboelectric sensors utilizing ion-conducting polymers (such as hydrogels, organogels or ionogels) as the electrodes have been reported recently achieving low modulus and high biocompatibility [21, 22], while it is still challenging for these iontronic triboelectric sensors to achieve high outputs at subtle mechanical disturbances and to demonstrate the combined advantages of softness, stretchability and breathability.

In this article, we propose an all-fiber iontronic triboelectric artificial mechanoreceptor that can satisfactorily fulfill these requirements. This ITM is comprised of electrospun dielectric polymer fibers and conductive ionogel fibers, and it can be ultrathin (average thickness of ~ 2.5 μm), ultralight (0.076 mg cm^−2^), breathable, stretchable and skin-compliant, making it imperceptive when mounted onto human skin. On the one hand, efficient mechanical-to-electrical energy conversion is obtained by achieving a high-output instantaneous specific power density; on the other hand, the ITM exhibits attractive sensitivity when serving as epidermal sensors. The ITM is demonstrated viable to perform precise health status monitoring, calibrating by detection of physical activities and vital signals such as radial pulse of human bodies. Besides, similar to the basilar membrane in the human cochlea, acoustic-to-electrical conversion by the ITM is further explored, and biometric application such as the noise dosimeter is successfully demonstrated. Moreover, to prove its stability when working on human skin, the performance of the ITM is investigated under various mechanical deformations, in order to mimic the scenarios in human daily activities, and the ITM performance is also evaluated under extreme humidity/perspiration conditions, to emulate the skin sweating situations. The desirable advantages in imperceptible and breathable configuration as well as the versatilities of the ITM make it highly promising for next-generation advanced epidermal electronics and prosthesis.

## Experimental Section

### Materials

Thermoplastic polyurethane (TPU) particles (Elastollan, 1180 A) were purchased from Sigma-Aldrich Chemical Co. Ltd. Dimethylformamide (DMF, 319937), and the ionic liquid [EMIM][TFSI] was purchased form Aladdin Chemical Co. All purchased chemicals were of analytical purity. Ltd. Deionized (DI) water was used in all of the experiments.

### Fabrication of the ITM

The ITM is fabricated by two-step electrospinning technology: (i) to prepare the precursor solution of ionic conductor electrode. TPU particles were dissolved in DMF at concentration of 10 wt%, and then stirred for about 12 h. [EMIM][TFSI] with certain mass ratios was then added to prepare the ionic gelatinous polymer. (ii) The electrospinning process was performed using an electrospinning device (Ucalery, ET-2535H). The precursor solution was loaded into a syringe capped with a 23-gauge metal needle at a constant feed rate. A high voltage of 20 kV was applied to the needle tip. The resultant nanofibers were deposited onto the aluminum foil-covered grounded metallic rotating roller at a rotation rate of 80 rpm with a 15-cm spinneret-collector distance. (iii) The precursor solution of TPU triboelectrification layer was prepared by dissolving TPU into DMF solvent at concentration of 10 wt% and then stirred for about 12 h. The applied electric voltage of 10 kV was applied by a high-voltage supply at the tip of the 23-gauge syringe needle. The TPU nanofibers were electrospun directly on the above electrode layer. (iv) After electrospinning process, the ITM was dried overnight under vacuum at room temperature to remove the residual solvent and then was carefully peeled off from the aluminum foil. The thicknesses control for the nanofibers was achieved by changing the spinning time.

### Evaluation of Water Vapor Permeability

The water vapor permeability was evaluated by measuring the weight loss of water permeated through the nanofibers film. Twenty grams of DI water was placed in a glass bottle with an opening aperture (1 cm in diameter). The glass bottles were covered by three different nanofibers film, *i.e.*, pure ionic electrode layer, pure TPU nanofibers layer, and the ITM layer. Then, the bottles were left undisturbed in a chamber at 25 °C and 30% humidity for 10 days. For comparison, the control group was covering nothing film and was exposed to the air. The results showed that the ITM exhibits excellent permeability (Fig. S3).

### Characterizations and Measurements

The surface morphology was characterized by field emission scanning electron microscope (FESEM, Hitachi SU8020). The optical transmittance of the samples was measured by a UV–Vis NIR spectrophotometer (Shimadzu, UV-3600). The thickness of the nanofiber film was measured by a step profiler. In this report, the measured diameter for the pure TPU nanofiber is around ~ 610 nm, and the diameter for the ionic electrode nanofiber (with 60% ionic liquid) is around 150–210 nm. The conductivity was measured by an electrochemical work station (CHI 660E). The electric resistance of the ionic electrode layer was measured by a Keithley electrometer 2450. The thermal stability test of the electrode layer at different temperatures was conducted in a thermostat oven (GDW-50L, Wuxi Zhongtian Company). Mechanical property was conducted using an ESM301/Mark-10 tester under a constant speed of 100 mm min^−1^. For the measurement of electric output performance, a step motor (LinMot E1100) was used to provide the input of mechanical motions. For all the tests of energy generation of the ITM, the pressure (100 kPa) and frequency (~ 2 Hz) of the step motor were fixed, and the device areas were fixed as 3 × 3 cm^2^. And the conductive fabric was used as the conductive wire for electrical characterization. The open-circuit voltage, short-circuit current and transferred charges were recorded by a programmable electrometer (Keithley electrometer 6514). For the monitoring of the radial pulse, the current signal was recorded with a Stanford low-noise preamplifier SR570. For the acoustic measurement, a sound box (Newmine K97) and the frequency modulation module RC-127 were used for modulating the audio signals. The software platform was constructed on the basis of LabVIEW, which is capable of realizing real-time data acquisition control and analysis.

## Results and Discussion

### Ultrathin, Ultralight and Breathable Iontronic Triboelectric Mechanoreceptor

**Fig. 1:**
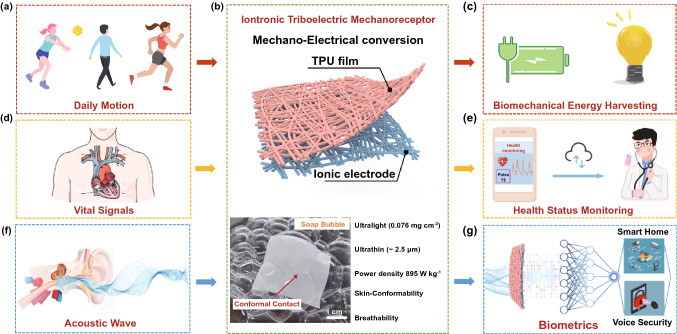
Overall concept of ITM for multi-functional epidermal electronics enabled by mechano-electrical conversion. **a**, **c** Proposed images of the ITM for biomechanical energy harvesting; **d**, **e** the ITM is used for health status monitoring (cardiovascular monitoring) and the future vision for intelligent wearable health monitor; **f**, **g** highly sensitive frequency–response ITM mimicking the basilar membrane of human cochlea to achieve intelligent voice user interfaces through collaborating with machine learning algorithms; **b** basic structure scheme of the ITM (top); photograph of the ultrathin and ultralight ITM floating on fragile bubbles (bottom)

**Fig. 2:**
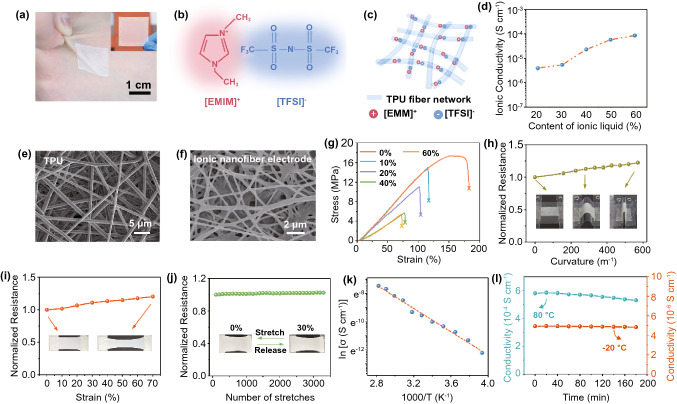
Electro-mechanical properties of ionic nanofiber electrode. **a** Photograph of the ITM attached on the human hand; **b** chemical structural formula of ionic liquid ([EMI][TFSI]); **c** conductive mechanism of the ionic nanofiber electrode based on TPU scaffold and the ionic liquid; **d** influence of EMITFSI concentration on the conductivity of the ionic nanofiber electrode; **e**, **f** SEM images for pure TPU nanofibers and the ionic nanofiber electrode (ionic liquid of 60 wt%); **g** stress–strain curves when increasing concentration of EMITFSI in ionic nanofiber electrode; **h** electric resistance of the ionic nanofiber electrode at varying bending curvatures from 0 to 570 m^−1^; **i** electric resistance of the ionic nanofiber electrode as a function of uniaxial tensile strain up to 70% stretching; **j** cyclic stretching test of the ionic nanofiber electrode at strain of 30%; **k** Arrhenius ionic conductivity (σ) plots of the ionic nanofiber electrode; **l** thermal stability of the ionic nanofiber electrode at both low (− 20 °C) and elevated (80 °C) temperatures

Figure 1 schematically illustrates the overall concept of the ITM for biomechanical kinetic energy harvesting, and for monitoring human activities, physiological signals or acoustic waves. The essence of the ITM is to realize mechano-to-electrical energy conversion (Fig. 1b). When the mechanical input is energetic like the daily human motions, the generated electrical output is potentially viable for power supply (Fig. 1a, c), whereas if the mechanical stimuli is subtle like the vasodilation or sound vibration from human body, the ITM is still able to output electrical signals potentially feasible for health monitoring (Fig. 1d–e) or biometric applications (Fig. 1f–g). Requirements including breathability, high skin-compliance and mechanical imperceptibility are crucial preconditions for epidermal devices in order to promote comfort and durability throughout the long-term wearing. The soft ITM has fully satisfied these expectations by adopting the structure of a bilayer nanofiber networks (Fig. 1b). The double-layer structure comprises the pure TPU nanofibers and the ionic conducting nanofiber electrode, both of which are prepared via a facile electrospinning strategy (Fig. S1). The yielded ITM has a weight of 0.076 mg cm^−2^ (*i.e.*, 0.68 mg for area of 3 × 3 cm^2^) with an average thickness of ~ 2.5 μm (Fig. S2), so it allows completely intimate conformal contact even with the fragile soap bubbles (see the photograph in Fig. 1b). The open and porous geometry endows the ITM with excellent breathability (as confirmed by the water evaporation measurement in Fig. S3), which is ensured by the three-dimensional micro-/nano-hierarchical pores in the bilayer interlaced nanofiber networks. This air permeability can facilitate heat and moisture exchange of the micro-environment between human skin and outer atmosphere, so as to minimize perturbation and physical constraints to the natural skin. Figure 2a presents the photograph of an ITM membrane, which exhibits decent optical transparency (~ 70%, see Fig. S4). Assisted by a VHB tape, the ITM membrane could be attached onto the forearm, resulting in a seamless and conformal contact with the human skin. More importantly, the ITM membrane could follow a large deformation of the skin without delamination (Fig. 2a).

### Properties of the Ionic Nanofiber Electrode

The intrinsically stretchable electrodes with excellent robustness are pivotal for rendering measurement precision and stability during unpredictable human motions. As all know, conventional commercial or laboratory-level electronic devices usually employ electrons as signal carriers; while living tissues rely predominantly on ions for signals propagation. In practice, in order to stimulate the biological systems, the electrons flows have to be converted to ion current at the electrode/biology interfaces. Inspired by this, we here adopted ionic conductors as electrodes in the ITM. The ionic nanofiber electrode comprises TPU nanofiber scaffold with ionic liquid (1-Ethyl-3-methylimidazolium bis(trifluoromethylsulfonyl)imide, [EMI][TFSI]) (Fig. 2b) confined inside the polymer chain networks. TPU here is chosen as the polymer scaffold for rendering device durability, since it can offer desirable mechanical properties and strength. As listed in Table S2, we compare the mechanical properties of 14 types of nanofibers film based on electrospinning technology. Taking the critical factors into consideration (including mechanical tensile strength, Young’s modulus and maximum elongation strength), TPU exhibits superior mechanical properties among the most common used polymer nanofibers, manifesting its feasibility in robust epidermal applications. Meanwhile, the polymer chain network of TPU offers a homogeneous medium allowing highly mobile ions confined within the nanofibers, thus assuring the credible conductivity (Fig. 2c). The ionic conductivity and mechanical properties of the ionic electrodes are highly related to the concentration of the ionic liquid. The conductivity at room temperature (25 °C) approaches 8.57 × 10^−5^ S cm^−1^ as the ionic liquid reaches 60 wt% (Fig. 2d). A morphology transition was observed from independent nanofibers structure (pure TPU nanofibers, see Fig. 2e) to a cohesive nanofibers formation in the polymer network (60 wt% [EMI][TFSI], see Fig. 2f); it was also observed that the addition of ionic liquid favors the thinner nanofibers, which was consistent with previous research [23].

Epidermal devices must be able to withstand large deformations (> 50% strain) for implementing various human activities in daily life [10, 24]. We therefore measured the mechanical property of the ionic electrodes membranes. With the [EMI][TFSI] content increasing from 0 to 60 wt%, the Young’s modulus, failure strain and ultimate tensile stress of the ionic electrodes all decreased (Figs. 2g and S5). Detailed explanations for this decrease are discussed in Fig. S5. But the capability for accommodating tensile strain beyond 60% is demonstrated for all these samples. The ionic electrode membrane with 60 wt% [EMI][TFSI] exhibited a Young’s modulus of 75.4 kPa, which is even lower than the human epidermis itself, ~ 100–200 kPa [25]. Next, the conductivity stability of the ionic electrode was investigated as it experienced different mechanical deformations, including bending and stretching. The ionic electrodes showed resistance increment of 22% as the bending curvature reached 570 m^−1^ (Fig. 2h) and 29% as the stretching strain reached 70% (Fig. 2i), respectively. To facilitate the bending measurement, the ionic electrode was placed onto a polyethylene terephthalate (PET) film for testing (inset in Fig. 2h). Noticeably, the ionic electrode could maintain great conductivity against cyclic strain tests, as supported by the experimental data in Figs. 2j and S6. The relative resistance of the electrodes barely changed, for increasing only 2.5% (under 30% strain), 5% (under 40% strain) and 7% (under 60% strain) after being stretched over 3000 cycles, respectively. Moreover, we also measured the conductivity variations of the ionic electrode after storing 3 weeks under room temperature (Fig. S6). Also, the thermal stability of the ionic electrode was further studied at both low (− 20 °C) and elevated (80 °C) temperatures. Experimental results showed that the ionic electrode was quite stable when storing at both temperatures, the ionic conductivity barely changed after 3 h (increase of 8.6% at 80 °C and 1.8% at − 20 °C), as exhibited by the data in Fig. 2l. Also, an improved ionic conductivity was obtained as the temperature rising from − 20 °C (4.92 × 10^–6^ S cm^−1^) to 80 °C (5.70 × 10^–4^ S cm^−1^), following well with the Arrhenius equation with an activation energy of 0.36 eV (Fig. 2k). According to previous report, this can be attributed to the fact that elevated temperature leads to more intense movement of polymer chains and ions, thus resulting in higher conductivities [23].

### High Specific Power Density for Mechanical Energy Harvesting

**Fig. 3:**
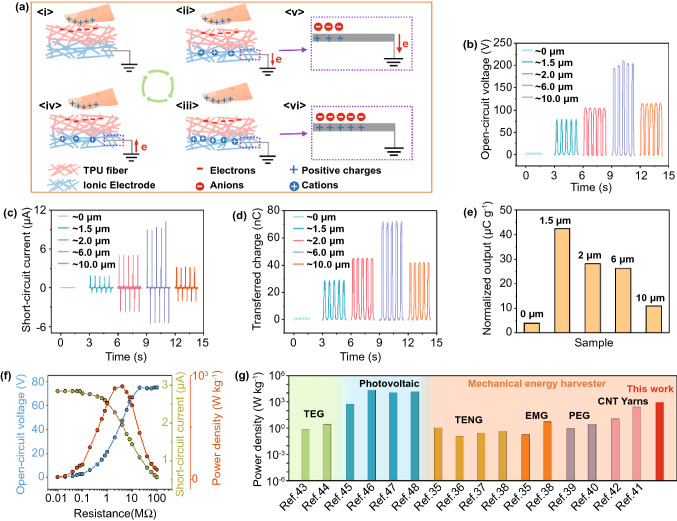
The operation principles and output performance of the ITM. **a** Working mechanism of the ITM when contacts with human finger; **b**–**e** with ionic electrode layer fixed at 1 μm, the open-circuit voltage, short-circuit current, transferred charges and normalized output of the ITM when increasing the thickness of TPU layer; **f** with the ionic electrode layer of 1 μm and the TPU layer of 1.5 μm, open-circuit voltage, short-circuit current and power curves with external resistance ranging from 0.01 to 100 MΩ; **g** comparison of weight specific power density between the ITM and other environmental energy technologies, including mechanical energy harvesters (TENG, EMG, PEG, and electrochemical harvester based on CNT yarns), thermoelectric generator and photovoltaic devices. *TEG* thermoelectric generator, *TENG* triboelectric nanogenerator, *EMG* electromagnetic generator, *PEG* piezoelectric generator

Adaptive and sustainable power supply is always the dilemma of epidermal electronics toward prolonged operation. Batteries offer the promising options, but they are still severely hindered by the weight/size and periodic recharging demands [26–28]. Ideally, the human body provides a wealth of potential energy sources: kinetic energy (such as body activities and muscle stretching), feeble vibrational energy (such as heartbeat, acoustic and pulse vibrations), hydraulic energy (such as human biofluids and blood flow) and chemical energy (such as glucose) [29, 30]. Thus directly harvesting energy from human body can be an effective strategy for powering epidermal electronics. Triboelectric nanogenerator (TENG), which is derived from Maxwell's displacement current, has been demonstrated efficient in scavenging dispersed, weak and low-frequency biomechanical energy [31]. The ITM here adopts a single-electrode triboelectric nanogenerator (S-TENG) design, leading to an overall ultralight (0.68 mg for area of 3 × 3 cm^2^) and ultrathin (average thickness of 2.5 μm) entity. The working principle of our ITM can be elaborated by referring to the basic model of a S-TENG with ionic electrodes, *i.e.*, coupling the mechanisms of contact electrification and electrostatic induction to generate electric signals [32]. As reflected in Fig. 3a, when an external object (finger for illustration) contacts with the ITM, triboelectric charges are generated with negative ones on the ITM side, and positive ones on the human finger. Once the finger is moving away, the constant static charges quantity on the ITM surface will result in positive ions migration in the electrode layer underneath to balance the static charges (Fig. 3a-ii and iii). Meanwhile, the same amount of negative ions is formed at the interface of the electrical double layer between the metal wire/ionic electrode, leading to the electrons transfer from the metal wires to the ground through the external circuits due to electrostatic induction effect (Fig. 3a-v and vi). When the finger is approaching again, the overall process will be reversed and a flow of electrons will transfer from the ground to the metal/ionic electrode interface (Fig. 3a-iv).

Structure parameters of the ITM have been optimized to obtain the desirable output. The contact pressure was 100 kPa, and contact area for the testing was 3 × 3 cm^2^, and the PTFE film was utilized as the external triboelectrification film to evaluate electrical outputs of the ITM. Detailed experiment parameters are demonstrated in Experimental Sections. It was found that with the ionic electrode layer fixed at ~ 1 μm, there exists a trade-off between the TPU layer thickness and the output performance of the ITM. The typical output parameters, including measured open-circuit voltage (${V}_{OC}$), short-circuit current (${I}_{SC}$) and transferred charges (*Q*) of the ITM, substantially increased with the rising thickness of TPU layer firstly, and reached the maximum value when TPU layer was ~ 6 μm, then declined with the TPU layer further thickened (Fig. 3b–d). This can be explained by that the appropriate increase of triboelectrification layer thickness is beneficial to larger contact area so as to boost the triboelectric charge generation, whereas excessive thickness erodes the electrostatic induction effect and thus the quantity of electron flow [33]. It is worth to be note that when the thickness of TPU layer is 0 μm, the ITM also shows a very weak output, which is due to the fact that the ionic nanofiber electrode can act as both the electrode layer and triboelectrification layer itself. For the TENG, the transferred charge *(Q)* is a critical criterion in evaluating the output performance [34]. Hence, the normalized output for ITM, which is defined as the amount of *Q* per gram, is presented in Fig. 3e. Likewise, the investigation to optimize the thickness of the ionic electrode layer is demonstrated in Fig. S8. As a result, when the thickness of the TPU layer is ~ 1.5 μm and the ionic electrode layer is ~ 1 μm, the weight of the whole ITM entity is 0.68 mg, and the optimal weight specific value is calculated to be 42 μC g^−1^; this ITM membrane is therefore chosen as the optimal sample. Next, the variations of current, voltage and output power density of the optimal sample with external resistance ranging from 0.01 to 100 MΩ were further measured, and an instantaneous power density (895 W kg^−1^) was obtained under the external resistance of 4 MΩ (Fig. 3f). This specific weight power density even holds advantages when compared to that of state-of-the-art environmental energy harvesters [35–42]. Selected representative reports are summarized in Fig. 3g and Table S1. We compared various mechanical energy harvesters, including the TENGs, the electromagnetic generators (EMGs), the piezoelectric generators (PEGs) and the harvesters based on carbon nanotube (CNT) yarns. The result implies the high output of our ITM in mechanical energy harvesting, thanks to the lightweight structure and the high electric output. In addition, even referring to other cutting-edge environmental energy technologies, *i.e.*, thermoelectric generators (TEGs) and photovoltaic devices, the ITM also remains competitive performance [43–48]. More advantages and differences between the proposed ITM with other triboelectric mechanoreceptors are discussed detailedly in Supporting Information (Note 1). The desirable result renders that our ITM is promising in high-performance self-powered epidermal electronics.

### Noninvasive Health Status Monitoring

**Fig. 4:**
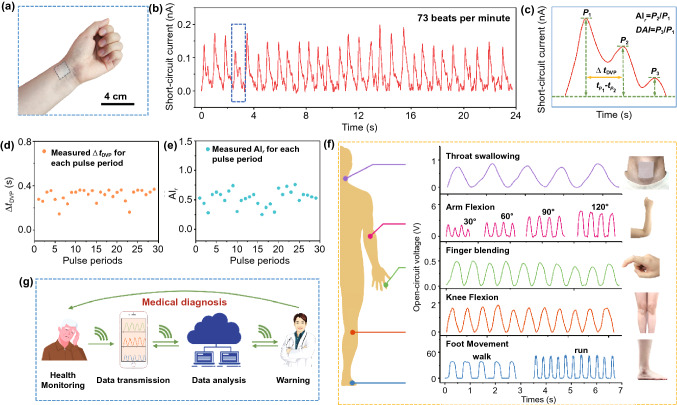
Health status signal monitoring by the ITM. **a** The ITM was attached onto the wrist for pulse detection; **b** the 25-s real-time and continuous monitoring of the pulse signals from the ITM; **c** one enlarged pulse signal from the pulse periods. **d**, **e** ∆${t}_{DVP}$ and ${AI}_{r}$ derived from 29 measured complete pulse periods; **f** the ITM for whole-body activities monitoring; **g** illustration of next-generation intelligent mobile diagnosis system

Enabled by the excellent mechano-to-electrical conversion ability, the feasibility of the ITM in human health status monitoring is also validated, calibrating by the physical activity and vital signs monitoring. The pulse waveforms stemming from radial artery contain critical indicators referring to cardiovascular problems, which are closely related to hypertension, arteriosclerosis, and diabetes diseases [9]. It’s not easy to capture the arterial pulse due to the imperceptible degree of deformations in the wrist skin, where the corresponding pressure level is less than 0.6 N cm^−2^ [49]. We here demonstrate the capability of the ITM for noninvasively detecting the vital information encoded in the arterial pulse with high fidelity, with it attached onto the wrist just above the radial artery (Fig. 4a). Figure 4b shows a real-time record over several pulse periods which contain detailed features, and the heart rate of the wearer is determined as 73 beats per minute. It was observed that the exact peaks for each cycle are not the same, which can be explained by that, in order to mimic the practical usage scenario as closely as possible, the signals were detected in the open and dynamic environment. Therefore, the undulations of the measured P1, P2 and P3 arose from the environmental disturbances. The selected magnified pulse readout of the periods is shown in Fig. 4c, where three distinct peaks (P1, P2 and P3) are clearly observed. P1 (pulse pressure) is the difference between systolic and diastolic blood pressure and is resulted from the blood flow ejected by heart contraction; P2 and P3 are blood reflections from the lower body and from the closed aortic valve, respectively [50]. These feature points are significant reflections of human health status, involving arterial stiffness, peripheral resistance, and left ventricular contractility associated with the cardiovascular problems. Although many epidermal technologies are reported inability to detect the weak peak information of P3, our ITM shows attractive capacity to pick up the tiny signals in the diastolic tail of the pulse peak with high fidelity, indicating huge potential in more accurate diagnosis [51].

The shape of artery pulse waveforms is affected by arterial stiffness, pulse wave velocity and wave reflections [50, 51]. From the three peaks (P1, P2 and P3), one can easily derive the typical criterions for arterial stiffness assessment, that is, the time delay between the first two peaks ($\Delta {t}_{DVP}$), the radial augmentation index (${AI}_{r}$= P2/P1), and the radial diastolic augmentation index ($\mathrm{DAI}$ = P3/P1). As plotted in Fig. 4c, the calculated values of ${\Delta t}_{DVP}$, ${AI}_{r}$ and $\mathrm{DAI}$ are 356 ms, 0.59 and 0.31, respectively, which are compliance with the health standards of 27-year-old males [52]. Moreover, to validate the precision and consistency of our ITM in cardiovascular monitoring, the detailed analysis toward the full 29 pulse periods in Fig. 4b was conducted. P1 and P2 are clearly observed for each period. The scatter diagrams of calculated ${\Delta t}_{DVP}$ and ${AI}_{r}$ (Fig. 4d, e) only demonstrate minute range fluctuations, indicating the consistency and accuracy of the ITM. Besides, although several data were missed, the majority of P3 peaks were successfully acquired with only small-scale fluctuations (see Fig. S9). The measured subtle signals highlight the suitability of our ITM in accurate and continuous cardiovascular diagnosis.

Considering that vital signals, such as heart rate and radial pulse, are strongly affected by the human activity; therefore, the physiological information alone is inadequate to access the health conditions of human beings. Aided monitoring of physical activities is also valuable because it provides much-needed and concrete context for the vital data, making it convincing for thorough analysis of wearers’ health condition and prevention/diagnosis of disease. Here we demonstrate the whole-body activities monitoring through the ITM. As exhibited in Fig. 4f, by attaching the ITM on different skin locations (throat, elbow, finger, knee, and foot), considerable voltage signals were detected referring to different motions. Notably, subtle signals caused by swallowing could be easily captured, and variable voltages under different movement frequency/amplitude of human being could also been distinguished. On the basis, the ITM is feasible in noninvasive health status monitoring which is calibrated by the health vital signals and the concrete activities detection. It is believed that our ITM has great promise when applied in future intelligent medical treatment, which is expected to implement health monitoring and diagnosis anytime and anywhere (Fig. 4g). Moreover, serving as on-skin or epidermal electronics will expose the ITM device to all kinds of mechanical stimuli such as friction, sliding, stretching or scratching. In this sense, stability concern of the ITM is the foremost issue need to be settled. Therefore, to mimic the daily physical activities and physiological conditions of human beings as closely as possible, we systematically investigated the stability of the ITM under various mechanical stimuli and under extreme humidity/perspiration conditions. Firstly, we tested the performance of the ITM under different deformations, including contacting, sliding, bending and stretching cycles, as shown in Figs. S7 and S10–S13. Consequently, the outputs maintain 68%, 70%, 90% and 86% of the initial value, after cyclic contacting (for 30,000 cycles), sliding (for 40,000 cycles), bending (for 30,000 cycles) and stretching (for 20,000 cycles) tests, respectively. Coupled with the SEM images of the ITM after all deformations, no nanofibers fracture is obtained, and consistent surface morphology is maintained under mechanical stimuli for tens of thousands times. Next, we measured the performance of the ITM under humidity and perspiration conditions, which are shown in Figs. S14 and S15. Results depicted that the ITM could keep decent output even under extreme humidity (over 90%) or under perspiration conditions, indicating its viability when applied as on-skin devices. After working over 3000 cycles under the perspiration condition, the output of the ITM is also 30% level of the initial value, and this performance is believed to be further improved through materials innovations or structure designs in future studies. The detail discussions are presented in Supporting Information.

### Acoustic Biometrics Applications

**Fig. 5:**
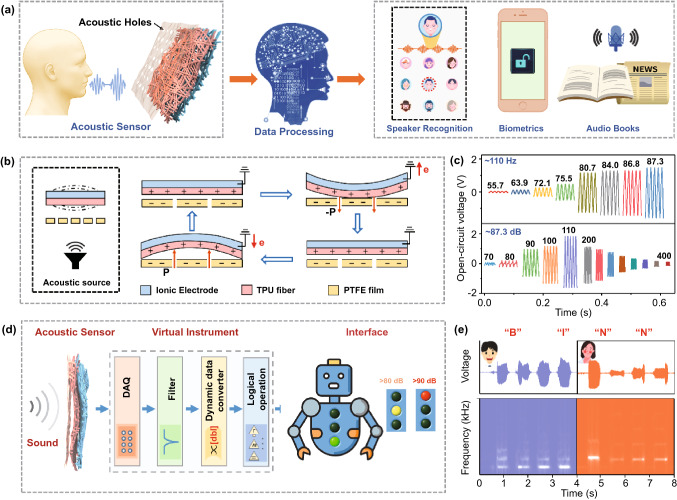
Acoustic biometrics applications of the ITM. **a** Expectation for intelligent biometric applications through the collaboration of the high-sensitivity ITM and the machine learning algorithms; **b** cross-sectional scheme for the working mechanism of the ITM during the vibration caused by sound waves; **c** voltage signal measured from the ITM when varying the acoustic frequency and SPL; **d** virtual sound detect robot interface for monitoring noise level; **e** voices recorded from a male and a female and the corresponding frequency spectrogram with simple letters (“B,” “I,” “N” and “N”)

The voice/speech is the most intuitive bio-signal for daily communication and information dissemination. Exploitation of the intelligent speech identification that emulates human auditory system is crucial yet challenging, and it involves two main parts, *i.e.*, advanced acoustic sensors and speech recognition algorithm. Acoustic sensors convert analog sound wave of human utterance into digital signals, and the converted signals can provide the test data for speech recognition software. Epidermal electronics require higher sensitivity and unique frequency–response feature to satisfy acoustic sensors needs. Here, the three-dimensional interlocked nanofiber networks geometry endows the ITM with superior sensitivity to other works (~ 165 mV sdB^−1^, Fig. S16), making it efficient in collecting sound waves over voice frequency range [53]. To facilitate the acoustic wave propagation, we implemented the experiment device in a Helmholtz resonant cavity during the measurement (Fig. S17). As indicated in Fig. 5a, a soft PTFE film with evenly distributed acoustic holes served as the external triboelectrification layer. The role of the PTFE film played in enhancing output performance and the detailed characterization of the PTFE film are depicted in Figs. S17-S18. Through interactions with the PTFE film, the ITM is able to monitor minute vibrations caused by sound pressure. Feasibility has been validated by previous research that through synergistic collaboration with machine learning (deep learning) algorithms, acoustic sensors can achieve voice user interfaces (UVI), such as speaker recognition, biometrics, virtual secretary, audio books and smart home appliances [54]. The working mechanism of the ITM for acoustic waves detection is based on the conversion of mechanical membrane vibrations caused by resonant sound waves into the electrical pulses, similar to that of basilar membrane in a human cochlea [54]. Figure 5b diagrammatically elaborates the detail process. When there exists an acoustic source, the wave trait of sound propagation causes periodic variations in the air pressure between PTFE film and the ITM, leading to periodic oscillation of the PTFE film. Thereafter, this deformation brings about the contact and triboelectrification between the PTFE film and the ITM. According to the difference in electron affinity, negative triboelectric charges are generated on surface the PTFE film, and the positive ones are on the ITM [14, 24]. When the air pressure varies, the PTFE film and the ITM get separate, resulting in a flow of electrons from ground to electrode in ITM driven by electrostatic induction. The returning contact between PTFE and ITM generates backflow of electrons.

To quantitatively depict the response of the ITM to acoustic waves, the dependence of open-circuit voltage on the sound pressure level (SPL) and frequency was investigated. We monitored the voltage waveforms of ITM in response to sound from a microphone. As illustrated in Fig. 5c, with the sound frequency fixed at 110 Hz, the voltage waveforms of ITM evidently increased with the rising SPL (from 55.3 to 87.3 dB). This is explained by the previous work that radial displacement of the PTFE film increases with the SPL, thus giving rise to an enhancement of open-circuit voltage [55]. It is worthy to note that sound under 60 dB is easily captured, which is the least magnitude of general speech signals on audible frequency range, revealing the superb sensitivity of the ITM [56]. And with a fixed SPL at 87.3 dB, the typical voltage signal of the ITM under the variable acoustic frequency is presented in Figs. 5c (bottom) and S19. Tested frequency ranges from 70 to 5000 Hz, which covers the basic frequency range of communication for human beings [51]. The output voltage waveforms exhibit a resonant feature and narrow output voltage peak, with a maximum voltage of 3.34 V (at 110 Hz), after which it gradually decreases (Fig. S19). Enabled by the high output over the baseline and unique frequency–response characteristic, we then explore the viability of the ITM working as a promising sound monitor. As all know, noise with SPL over 70–80 dB can cause discomfort of human beings, and noise with SPL beyond 90 dB can affect health status and even cause deafness. To this end, we develop a virtual sound detect robot interface based on a customized LabVIEW program to detect noise level, as reflected in Fig. 5d and Video S1. The robot is equipped with three indicator lights, each of which is switched by a calibrated threshold voltage. As such, when there exists sound wave, the output voltage of the ITM is monitored in real time, and appropriate-level indicator light will be turned on when the output voltage reaches corresponding threshold level. In this system, when the SPL exceeds 80 dB, indicator light will change to yellow from green; when it further increases to 90 dB, the light will change to red for intuitive warning. The whole process is highly responsive and fast.

More delicate data, voices from different human beings, were further monitored. Two speakers, a female and a male, were asked to repeat four English letters (“B,” “I,” “N,” “N”) in front of a standing microphone. The time-dependent voltages waveform and respective frequency domain signals analyzed by real-time fast Fourier transform (FFT) are exhibited in Fig. 5e. Evidently, the real-time peak spectrogram is retrieved, and the exclusive harmonic frequency of each letters is recorded accurately. Different letters demonstrate different frequency domains, and the low-frequency components of male spectrum are more prominent than female spectrum. Besides, time-dependent waveforms variations were also measured while the melody of “March of the Volunteers” (the national anthem of the People's Republic of China) was played. The ITM allows easy real-time voltages capture and display of the decoded frequency-domain information (Fig. S20). These results indicate the high applicability of the ITM as wearable or epidermal acoustic sensors, which holds great prospect in the coming era of artificial intelligence (AI) and Internet of Things (IoT).

## Conclusion

In summary, we demonstrate an all-fiber ITM for multi-functional epidermal electronics. This ITM is ultrathin (average thickness of ~ 2.5 μm), ultralight (~ 0.076 mg cm^−2^), breathable, stretchable and skin-compliant, and holds great stability under different mechanical deformations. Endowed with excellent mechano-to-electrical energy conversion ability, the ITM can satisfy various applications, including biomechanical energy harvesting, human activities monitoring, cardiovascular monitoring and acoustic biometric applications. Specifically, high-output instantaneous power density is obtained. Besides, the ITM can also perform health status monitoring, calibrating by detection of physical activities and vital signals of human pulse. Moreover, acoustic-to-electric signals conversion by the ITM is also demonstrated. The ITM can convert mechanical vibrations caused by sound waves into the electrical impulse signals, similar to the basilar membrane in a human cochlea. Delicate data including voices from different people and song melody were monitored through real-time voltages and the decoded frequency-domain information. And biometric application of the ITM as a noise dosimeter is successfully demonstrated. This ITM can fulfill multiple functionalities surprisingly, showing a promising prospect in applications of next-generation intelligent epidermal electronics.

## Supplementary Information

Below is the link to the electronic supplementary material.

Supplementary file1 (PDF 1234 kb)

Supplementary file2 (MP4 5714 kb)
