# Supplementary material for: Ultralight Iontronic Triboelectric Mechanoreceptor with High Specific Outputs for Epidermal Electronics
Source: Nanomicro Lett. 2022 Mar 29;14:86. doi: 10.1007/s40820-022-00834-4 (PMC8964870; doi:10.1007/s40820-022-00834-4)
Supplement: Supplementary file 1 — Supplementary file1 (PDF 1234 kb) [file 40820_2022_834_MOESM1_ESM.pdf]

Supporting Information for

Ultralight Iontronic Triboelectric Mechanoreceptor with High Specific Outputs for Epidermal Electronics

Hai Lu Wang[1, #], Zi Hao Guo[1, 2, #], Xiong Pu[1, 2, 3, 4, *], Zhong Lin Wang[1, 2, 5, *]

[1]Beijing Institute of Nanoenergy and Nanosystems, Chinese Academy of Sciences, Beijing 100083, P. R. China

[2]School of Nanoscience and Technology, University of Chinese Academy of Sciences, Beijing 100049, P. R. China

[3]Center on Nanoenergy Research, School of Physical Science and Technology, Guangxi University, Nanning 530004, P. R. China

[4]CUSTech Institute of Technology, Wenzhou, Zhejiang, 325024, P. R. China

[5]School of Materials Science and Engineering, Georgia Institute of Technology, Atlanta, GA 30332, USA

[#]Hai Lu Wang and Zi Hao Guo contributed equally to this work.

*Corresponding authors. E-mail: puxiong@binn.cas.cn (Xiong Pu); zhong.wang@mse.gatech.edu (Zhong Lin Wang)

Supplementary Figures

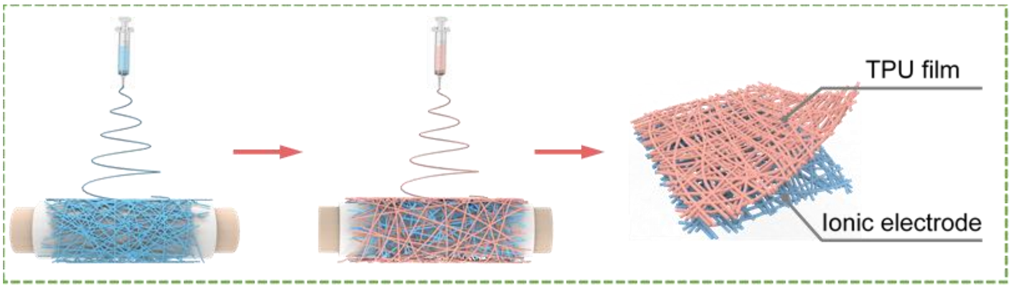

Fig. S1 The fabrication procedures of the ITM through electrostatic spinning process; The ITM is fabricated by two-step electrospinning technology; (i) The electrospinning process of the ionic electrode layer; (ii) The electrospinning process of the TPU layer. The TPU nanofibers were electrospun directly on the above electrode layer

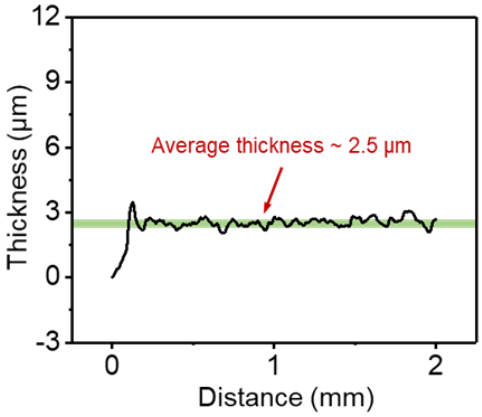

Fig. S2 Thickness characterization of the ITM; The thickness of the ITM membrane measured by the step profiler; The roughness of the surface is attribute to three-dimensional hierarchical stacked nanofiber networks

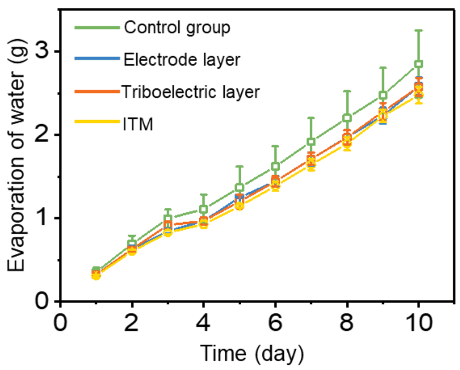

Fig. S3 Permeability property of the ITM; Water vapor permeability property of the pure TPU triboelectrification layer, ionic nanofibers electrode and the ITM membrane

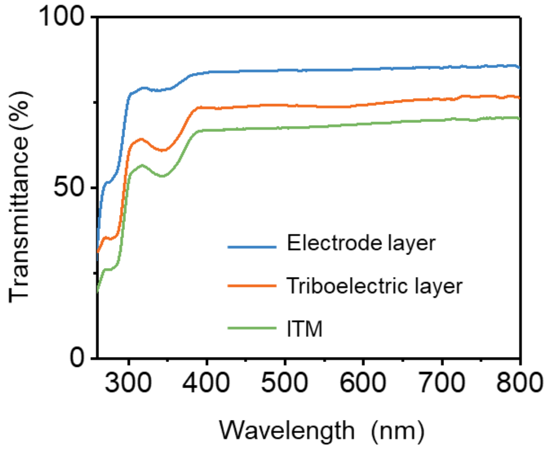

Fig. S4 Optical transmittance characterization; Optical transmittance spectra for the pure TPU triboelectrification layer, ionic nanofibers electrode and the ITM membrane

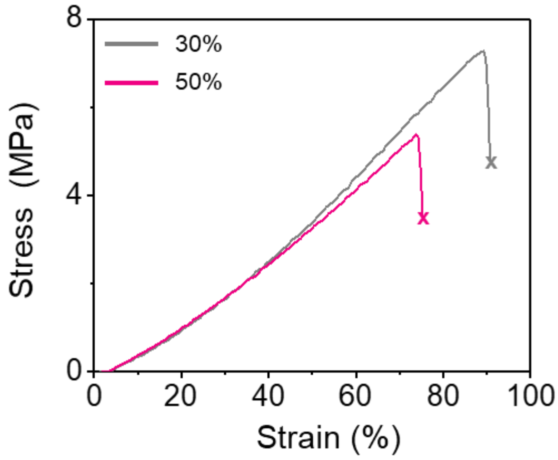

Fig. S5 Stress-strain curve; Stress-strain curve of the ionic nanofiber electrodes with [EMI][TFSI] concentration of 30% and 50%

It was observed that stress-strain curves decrease when increasing the concentration of EMITFSI in ionic nanofiber electrodes, and we ascribe the decreases to the finer nanofibers diameter. As shown in Fig. 2e-f, it was found observed that the addition of ionic liquid gives rise to the finer nanofibers. Under the same conditions for the electrospinning process, the resultant diameter for the pure TPU nanofiber is around ~610 nm, the diameter for the ionic electrode nanofiber (with 60% ionic liquid) is around 150~210 nm. In this regard, we infer that reduced mechanical strength of the ionic electrode film results from finer nanofibers.

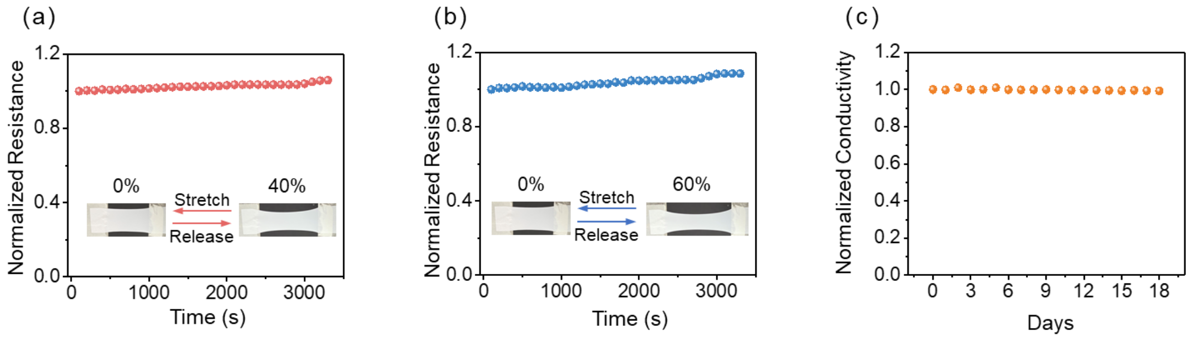

Fig. S6 Cyclic performance of the ionic electrode; **a** The relative resistance variations of the ionic electrode against cyclic strain at 40%; **b** The relative resistance variations of the ionic electrode against cyclic strain at 60%; **c** Conductivity variations of the ionic electrode after storage for three weeks at room temperature

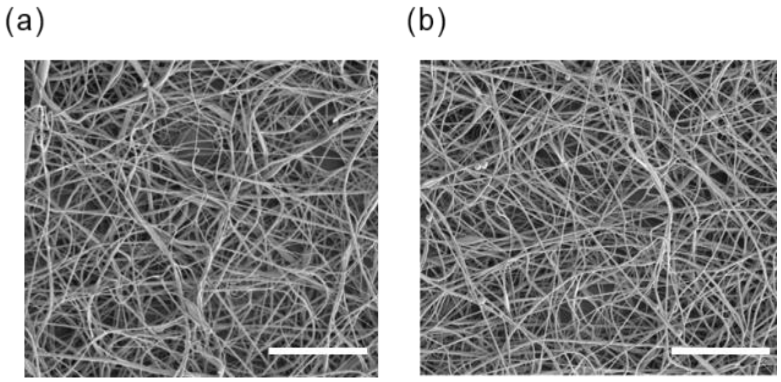

Fig. S7 SEM characterization of the surface morphology of the ITM; **a** SEM images for the initial surface morphology for the ITM; **b** SEM images for the ITM surface after being stretched over 20000 cycles (Scar bar is 50 μm)

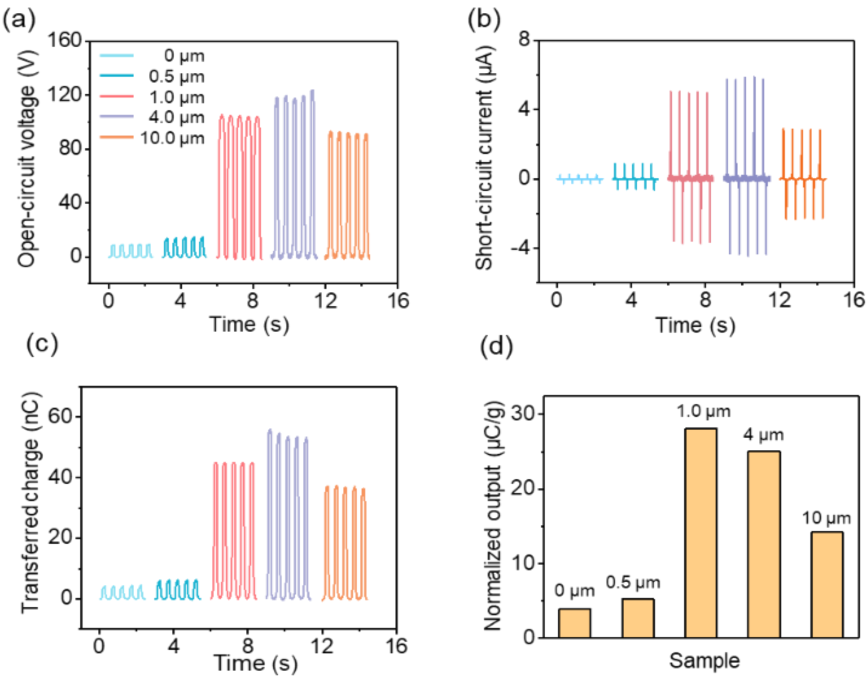

Fig. S8 The output performance of the ITM when the TPU triboelectrification layer was fixed at 2 μm; **a** Open-circuit voltage; **b** Short-circuit current; **c** Transferred charges and **d** Normalized output of the ITM when varying the thickness of the ionic electrode

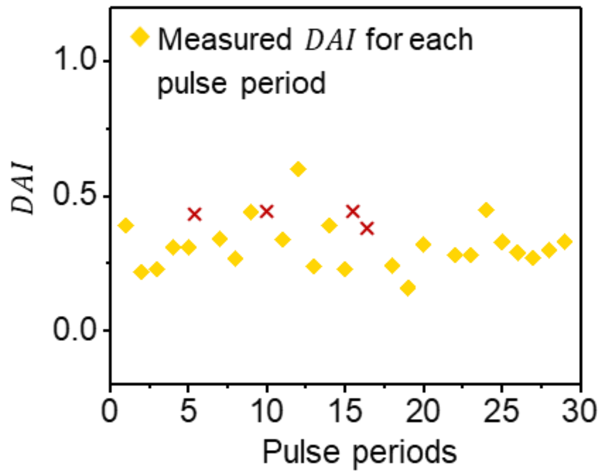

Fig. S9 Measured DAI; Measured DAI derived from the 29 complete pulse periods

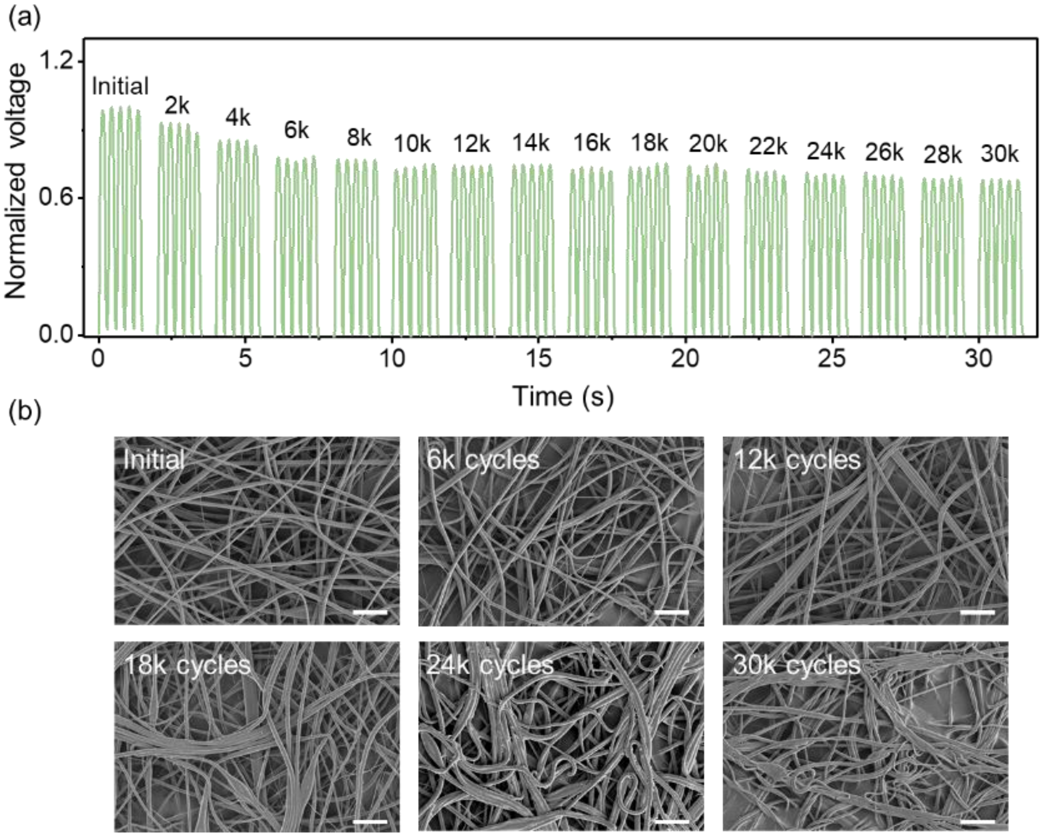

Fig. S10 Durability test of the ITM after contacting for 30000 cycles; **a** Open-circuit voltage of the ITM after contact-separation for 30000 cycles; **b** SEM images of the ITM surface after contact-separation cycles (Scar bar is 10 μm)

Figure S10 shows the open-circuit voltage of the ITM, and the SEM characterization of the ITM surface morphology after 30000 contacting cycles. Results showed that the output can maintain over 68% of the initial level after contact-separation for over 30,000 cycles. And the SEM images imply that, applied pressure caused by frequent contacts would make the nanofibers film become more tight and compacted, but no fiber fracture is observed after 30,000 contacts

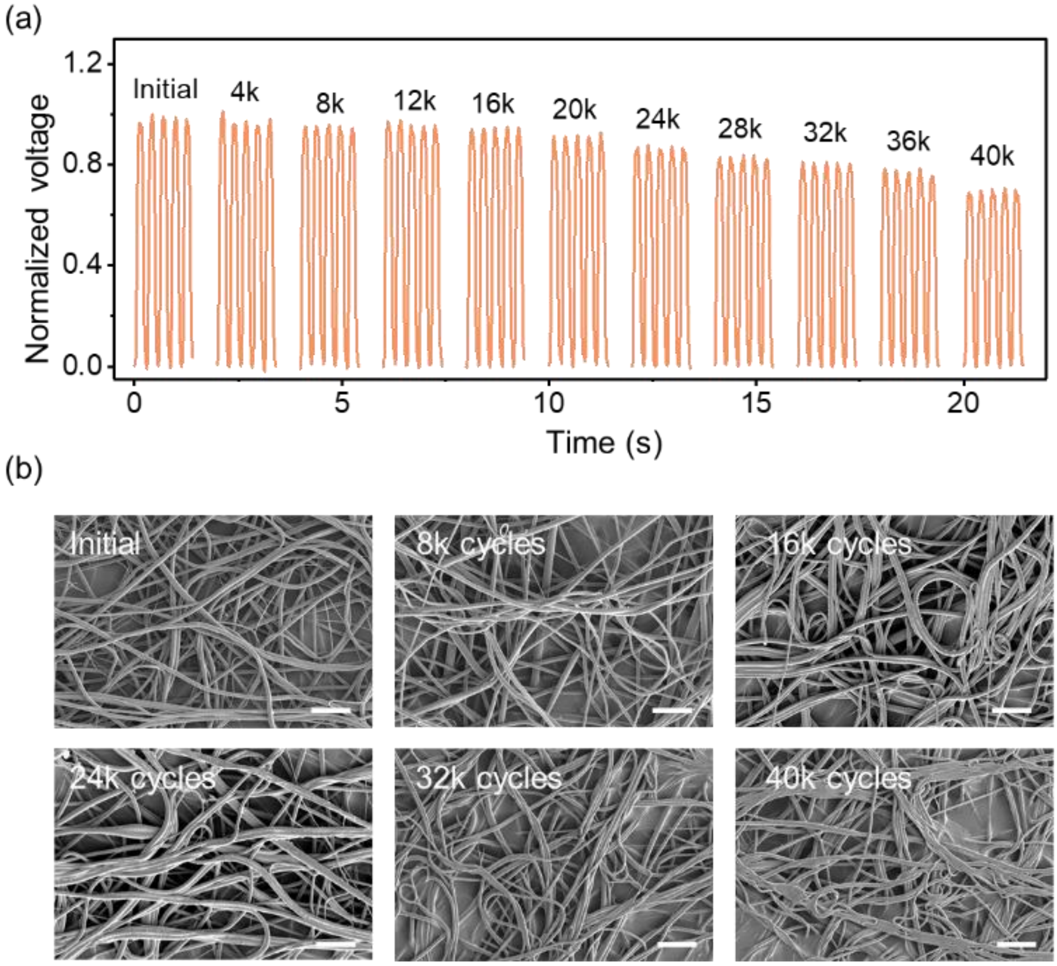

Fig. S11 Durability test of the ITM after sliding for 40000 cycles; **a** Open-circuit voltage of the ITM after sliding for 30000 cycles; **b** SEM images of the ITM surface after sliding cyclic test (Scar bar is 10 μm)

Figure S11 shows the open-circuit voltage of the ITM, and the SEM characterization of the ITM surface morphology after 40000 sliding cycles. Results showed that the output can maintain over 70% of the initial level after sliding for 40,000 cycles. Although showing a little attenuation, it's reasonably to believe that this output attenuation is acceptable and reasonable, since no encapsulation strategy was utilized in order to maintain the great breathability of our device. And due to the simple and cost-effective fabrication strategy, the ITM can also be used as disposable product for short-time usage. In this sense, the durability/stability for the ITM (under tens of thousands of cycles) is enough to support short-time (for one or several days) service. Indeed, we know that there's room for improvement of the ITM in stability and durability performance. And it's believed to be further optimized by materials modification and structure design in future explorations.

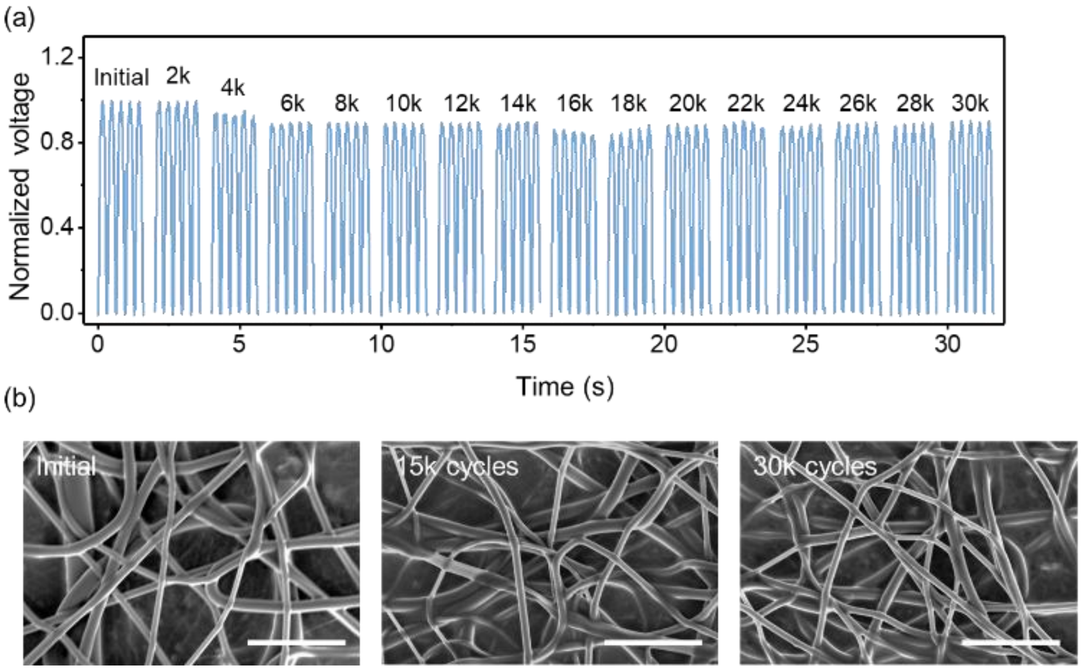

Fig. S12 Durability test of the ITM after bending for 30000 cycles; **a** Open-circuit voltage of the ITM after bending for 30000 cycles; **b** SEM images of the ITM surface after bending cyclic test (Scar bar is 10 μm)

Figure S12 shows the open-circuit voltage of the ITM, and the SEM characterization of the ITM surface morphology after 30000 bending cycles. Results showed that the output can maintain over 90% of the initial level after bending for 30000 cycles. And the SEM images imply no obvious morphology changes of the nanofibers film, and no fracture is observed in the fibers after 30000 bending tests.

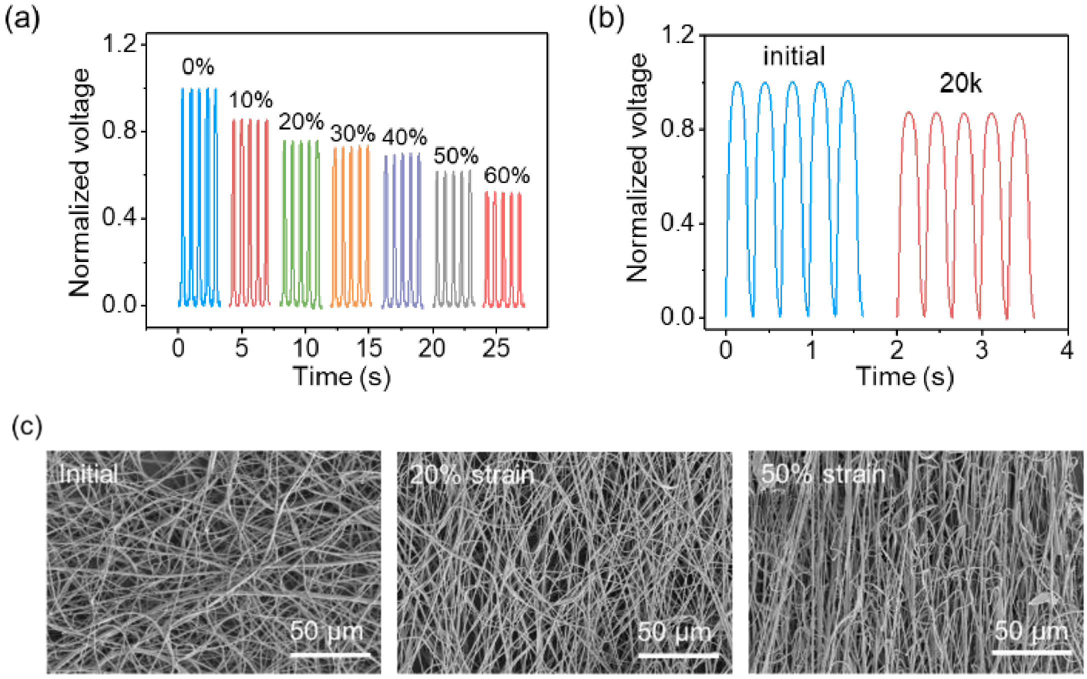

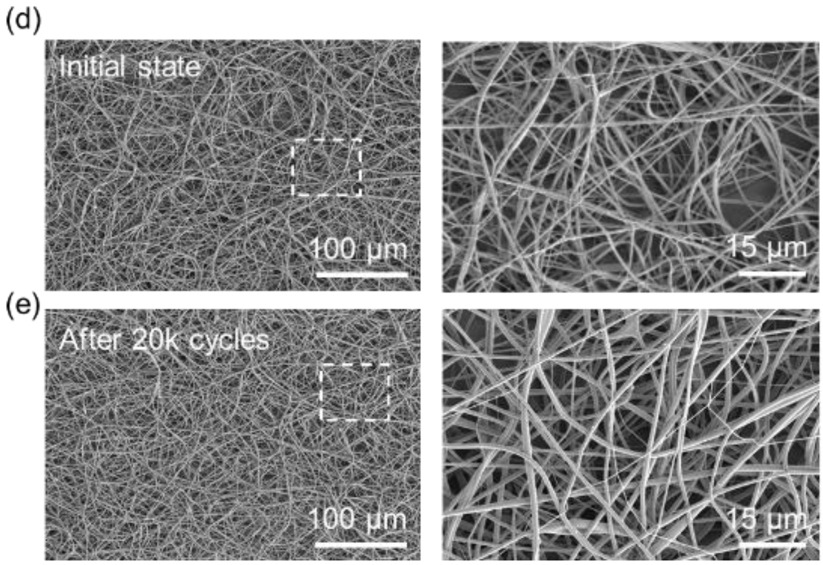

Fig. S13 Durability test of the ITM after stretching for 20000 cycles; **a** Open-circuit voltage of the ITM after stretching for 20,000 cycles; **b** SEM images of the ITM surface after stretching cyclic test

For the stretching cyclic tests, we measured the output performance of the ITM under different stretched states (from 0% to 60%), the open-circuit voltage can maintain 52% of the initial value when the ITM was stretched to 60% state (Fig. S13a). Then, we tested the performance of the ITM after stretching to 50% for 20,000 cycles, the output is 86% of the initial level, as demonstrated in Fig. S13b. SEM images in Fig. S13c demonstrate that, when the nanofiber film is stretched under external strain, the nanofibers are driven to directional alignment while no fibers fracture is observed. The contrastive SEM images of the ITM surface before (Fig. S13d) and after stretching (Fig. S13e) are consistent, indicating the great stability of the ITM.

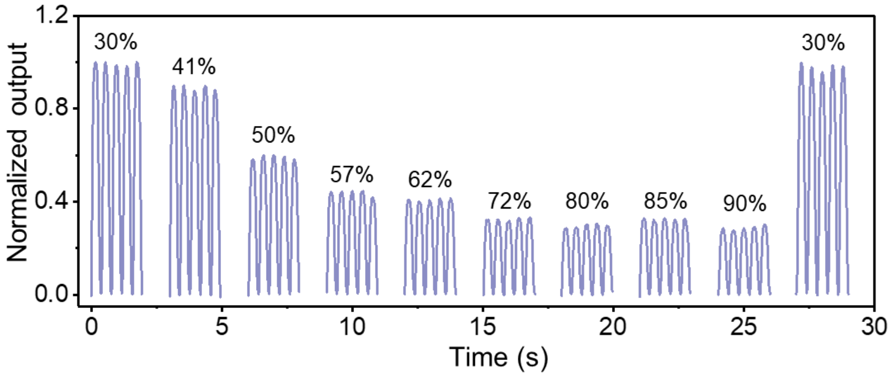

Fig. S14 Durability test of the ITM under humidity conditions; Open-circuit voltage of the ITM under different humidity conditions

Figure S14 presents the output of the ITM under different humidity conditions, from 30% to 90%. Since triboelectrification is mainly surface phenomenon, therefore open-circuit voltage of the ITM is distinctly affected by the environmental humidity and perspiration. But, the ITM also maintain over 28% of the initial value even under 90% humidity, and the output can recover to the initial value after the humidity recovered.

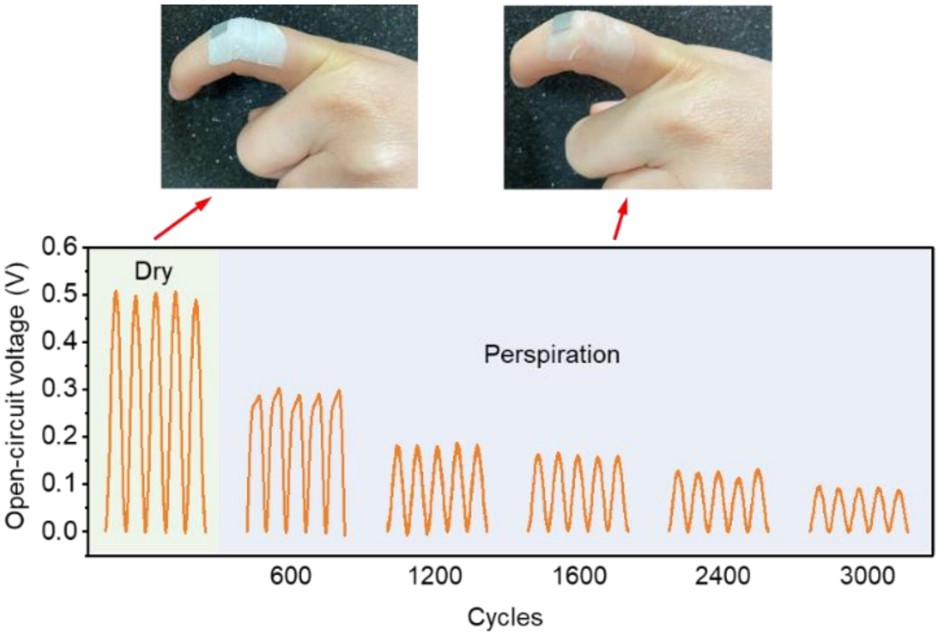

Fig. S15 Durability test of the ITM under perspiration conditions; The photographs of the ITM attached on the finger joint under dry and perspiration conditions (top); Cyclic output tests of the ITM under sweating conditions (down)

The output performance of the ITM under perspiration condition is shown in Fig. S15 Under sweating conditions, our ITM also demonstrates decent output after working for 3000 cycles. All these results prove that our ITM is suitable for on-skin and epidermal devices, it not only has the imperceptible and breathable structure, but also demonstrates excellent stability during daily activities.

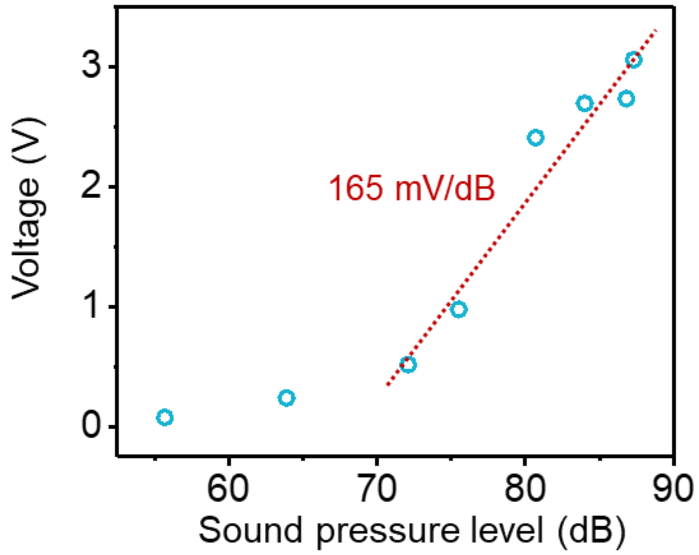

Fig. S16 Sensitivity of the ITM for acoustic energy sensing; The relationship between open-circuit voltage of the ITM and the sound pressure level (SPL)

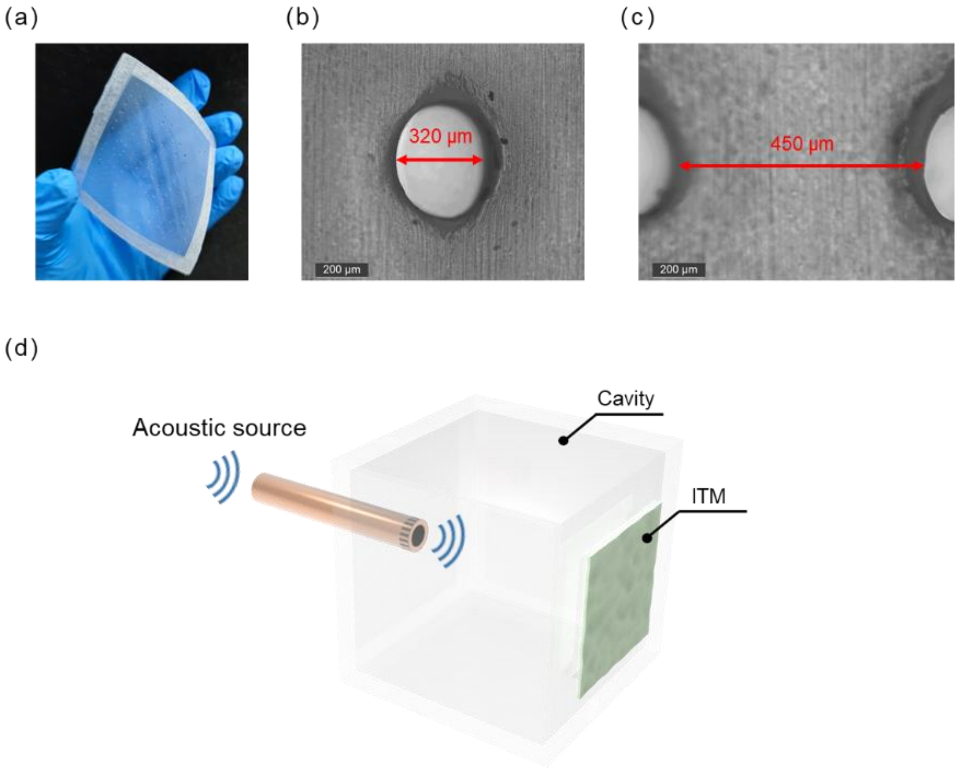

Fig. S17 Acoustic testing by the ITM; **a** Photograph of the PTFE film with acoustic holes distributed. (Scale bar: 1 cm); **b** Optical microscope photograph of the acoustic holes; **c** The gap between the acoustic holes; **d** Structure scheme of the Helmholtz resonant cavity for harvesting acoustic energy

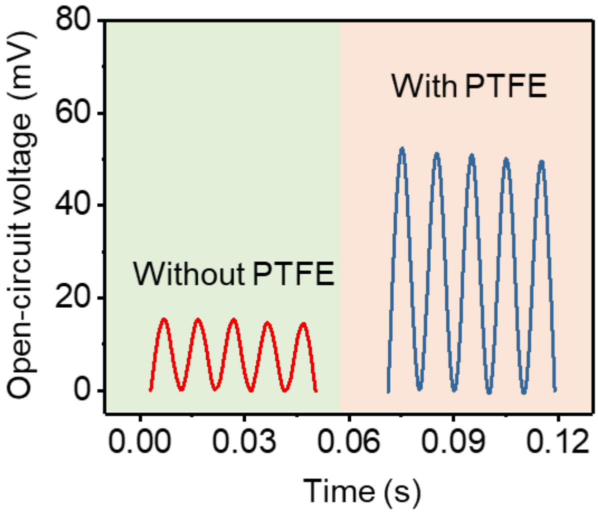

Fig. S18 Open-circuit voltage measured from the ITM with and without the PTFE film (with SPL of 55.7 dB and frequency of 110 Hz)

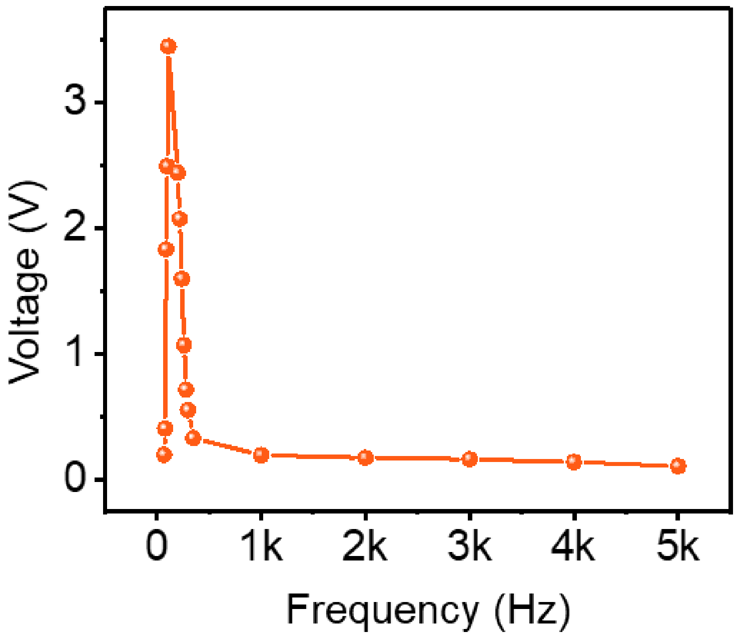

Fig. S19 Typical voltage signal of the ITM under the variable acoustic frequency; The relationship between open-circuit voltage of the ITM and the sound frequency (sweeping frequency ranges from 70 to 5000 Hz; sound intensity is 87.3 dB SPL)

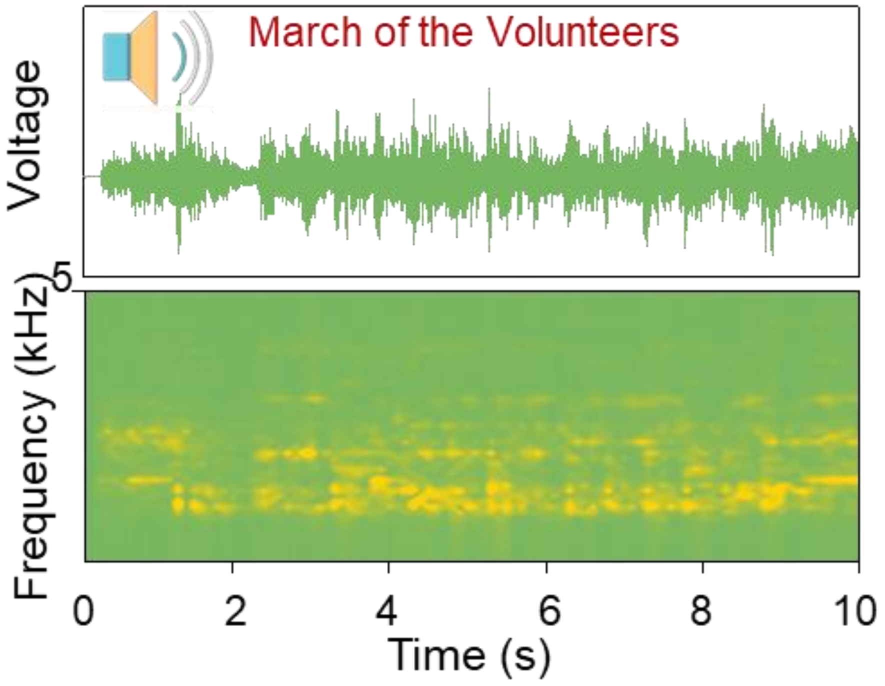

Fig. S20 Waveform and frequency spectrogram signals of melody "March of the Volunteers" extracted by the ITM; Real-time voltages capture and display of the decoded frequency-domain information

Table S1 Comparison of different environmental energy harvesters

**Energy harvesters**	**Materials/structure**	**Power density (W/kg)**	**Refs.**
TEG	carbon nanotube (CNT) fibers	1.9	[S1]
TEG	glass fabric	2.8	[S2]
TEG	carbon nanotube yarn	697×10^{-3}	[S3]
TEG	reduced graphene oxide sheets	4.19×10^{-3}	[S4]
Photovoltaic Harvester	perovskite solar cells	23×10^{3}	[S5]
Photovoltaic Harvester	organic photovoltaics	11.46×10^{3}	[S6]
Photovoltaic Harvester	PbS colloidal quantum dots	15.2×10^{3}	[S7]
Photovoltaic Harvester	nanocellulose paper-based perovskite solar cells	0.56×10^{3}	[S8]
PEG	PVDF/a multilayer and multistep configuration.	15.4×10^{-3}	[S9]
PEG	$0.71Pb(Mg_{1/3}Nb_{2/3})O_3$-$0.29PbTiO_3$	0.88	[S10]
PEG	PVDF/rGO/BT fibers	3	[S11]
Electrochemical Harvester	carbon nanotube yarn twist	0.25×10^{3}	[S12]
Electrochemical Harvester	hierarchically twisted carbon nanotube yarn	0.65	[S13]
Electrochemical Harvester	three-dimensional graphene aerogel	11.7	[S14]
Electrochemical Harvester	carbon nanotube yarn	5.3	[S15]
EMG	metal Cu and PA film/rotating-disk structure	24×10^{-3}	[S16]
EMG	Au electrode and PTFE film/ rotating-disk structure	180×10^{-3}	[S17]
EMG	metal Al and PA film/contact-separation structure	5.31	[S18]
TENG	Au electrode and PTFE film/ rotating-disk structure	1152×10^{-3}	[S17]
TENG	metal Cu and PA film/rotating-disk structure	119×10^{-3}	[S16]
TENG	Nylon and PVDF nanofibers/contact-separation structure	0.28	[S19]
TENG	metal Al and PA film/contact-separation structure	0.48	[S18]
**TENG**	**TPU/S-TENG of contact-separation structure**	**895**	**This work**

TEG: thermoelectric generator; PEG: piezoelectric generator; EMG: electromagnetic generator; TENG: triboelectric nanogenerator; S-TENG: single-electrode triboelectric nanogenerator; PVDF: polyvinylidene fluoride; rGO: reduced graphene oxide; BT: barium-titanium oxide; PA: polyamide; PTFE: polytetrafloroethylene

Table S2 Comparison of different polymer nanofibers based on electrostatic-spinning technology

**Materials**	**Young's Modulus (MPa)**	**Tensile strength (MPa)**	**Elongation at break (%)**	**Refs.**
Collagen	1.5	22	2	[S20]
CA	1170	12.1	1.31	[S21]
Nylon 6	19.4	10.45	250	[S22]
Nylon 6,6	20.9	6.5	140	[S23]
PET	60	3.7	-	[S24]
PCL	3.8	4.5	170	[S25]
PLA	8.7	0.76	-	[S26]
PMMA	12.9	0.3	-	[S23]
PU	45	4.5	-	[S27]
PVA	175	5.8	102	[S28]
PVC	12.3	2.2	90	[S23]
PVDF	168.9	3.7	-	[S29]
PVDF-HFP	10	10	-	[S30]
**TPU**	**13.3**	**17.34**	**183**	**This work**

Note 1:

To better convey the advantages of our ITM, the differences between the developed ITM and other reported triboelectric mechanoreceptors are listed as below:

1. The ITM can achieve versatile epidermal applications in single device. We demonstrate the ITM applying for mechanical energy harvesting, radial artery pulse monitoring, human activities monitoring, acoustic energy harvesting and biometric applications. More importantly, all these functions are performed in one single ultralight and breathable device. However, previous triboelectric mechanoreceptors are reported to achieve only one or two of these functions. For instance, some mechanoreceptors are unable to detect subtle pulse signals (with three distinct peaks), and others mechanoreceptors show inability for biometric applications.

2. The ITM shows high weight specific power density in harvesting mechanical energy. For previous mechanical energy harvesters, they need either sophisticated and bulk structure design or extra power management circuits to achieve desirable power density. [31-36] For our ITM, we achieve high-performance with an ultrathin and ultralight device. As summarized in Table S1, we compared the different energy harvesters. As a result, our ITM shows high weight specific power density compared with other mechanical energy harvesters; and also holds comparable performance advantages when referring to other cutting-edge environmental energy technologies.

Supplementary References

[S1] K.T. Park, T. Lee, Y. Ko, Y.S. Cho, C.R. Park et al., High-performance thermoelectric fabric based on a stitched carbon nanotube fiber. ACS Appl. Mater. Interfaces **13**(5), 6257-6264 (2021). https://doi.org/10.1021/acsami.0c20252

[S2] S.J. Kim, J.H. We, B.J. Cho, A wearable thermoelectric generator fabricated on a glass fabric. Energy Environ. Sci. **7**(6), 1959-1965 (2014). https://doi.org/10.1039/c4ee00242c

[S3] J. Choi, Y. Jung, S.J. Yang, J.Y. Oh, J. Oh et al., Flexible and robust thermoelectric generators based on all-carbon nanotube yarn without metal electrodes. ACS Nano **11**(8), 7608-7614 (2017). https://doi.org/10.1021/acsnano.7b01771

[S4] W. Zeng, X.M. Tao, S.P. Lin, C. Lee, D.L. Shi et al., Defect-engineered reduced graphene oxide sheets with high electric conductivity and controlled thermal conductivity for soft and flexible wearable thermoelectric generators. Nano Energy **54**, 163-174 (2018). https://doi.org/10.1016/j.nanoen.2018.10.015

[S5] M. Kaltenbrunner, G. Adam, E.D. Glowacki, M. Drack, R. Schwodiauer et al., flexible high power-per-weight perovskite solar cells with chromium oxide-metal contacts for improved stability in air. Nat. Mater. **14**(10), 1032-1039 (2015). https://doi.org/10.1038/Nmat4388

[S6] S. Park, S.W. Heo, W. Lee, D. Inoue, Z. Jiang et al., Self-powered ultra-flexible electronics via nano-grating-patterned organic photovoltaics. Nature **561**(7724), 516-521 (2018). https://doi.org/10.1038/s41586-018-0536-x

[S7] X.L. Zhang, V.A. Oberg, J. Du, J.H. Liua, E.M.J. Johansson, Extremely lightweight and ultra-flexible infrared light-converting quantum dot solar cells with high power-per-weight output using a solution-processed bending durable silver nanowire-based electrode. Energy Environ. Sci. **11**(2), 354-364 (2018). https://doi.org/10.1039/c7ee02772a

[S8] L. Gao, L.F. Chao, M.H. Hou, J. Liang, Y.H. Chen et al., Flexible, transparent nanocellulose paper-based perovskite solar cells. Npj Flex. Electron. **3**(1), 4 (2019). https://doi.org/10.1038/s41528-019-0048-2

[S9] R. Sriramdas, S. Chiplunkar, R.M. Cuduvally, R. Pratap, Performance enhancement of piezoelectric energy harvesters using multilayer and multistep beam configurations. IEEE Sens. J. **15**(6), 3338-3348 (2015). https://doi.org/10.1109/Jsen.2014.2387882

[S10] C.D. Xu, B. Ren, W.N. Di, Z. Liang, J. Jiao et al., Cantilever driving low frequency piezoelectric energy harvester using single crystal material $0.71Pb(Mg_{1/3}Nb_{2/3})O_3$-$0.29PbTiO_3$. Appl. Phys. Lett. **101**(3), 033502 (2012). https://doi.org/10.1063/1.4737170

[S11] F. Mokhtari, G.M. Spinks, S. Sayyar, Z.X. Cheng, A. Ruhparwar et al., highly stretchable self-powered wearable electrical energy generator and sensors. Adv. Mater. Technol. **6**(2), 2000841 (2021). https://doi.org/10.1002/admt.202000841

[S12] S.H. Kim, C.S. Haines, N. Li, K.J. Kim, T.J. Mun et al., Harvesting electrical energy from carbon nanotube yarn twist. Science **357**(6353), 773-778 (2017). https://doi.org/10.1126/science.aam8771

[S13] K.J. Kim, J.S. Hyeon, H. Kim, T.J. Mun, C.S. Haines et al., Enhancing the work capacity of electrochemical artificial muscles by coiling plies of twist-released carbon

nanotube yarns. ACS Appl. Mater. Interfaces **11**(14), 13533-13537 (2019). https://doi.org/10.1021/acsami.8b21417

[S14] X.S. Zhou, X. Chen, H. Zhu, X. Dong, L.Z. Li et al., Electrical energy generation by squeezing a graphene-based aerogel in an electrolyte. Nanoscale **13**, 8304 (2021). https://doi.org/10.1039/d1nr00544h

[S15] T.J. Mun, S.H. Kim, J.W. Park, J.H. Moon, Y. Jang et al., Wearable energy generating and storing textile based on carbon nanotube yarns. Adv. Funct. Mater. **30**(23), 2000411 (2020). https://doi.org/10.1002/adfm.202000411

[S16] X.D. Zhong, Y. Yang, X. Wang, Z.L. Wang, Rotating-disk-based hybridized electromagnetic-triboelectric nanogenerator for scavenging biomechanical energy as a mobile power source. Nano Energy **13**, 771-780 (2015). https://doi.org/10.1016/j.nanoen.2015.03.012

[S17] Y.D. Chen, Y. Cheng, Y. Jie, X. Cao, N. Wang et al., Energy harvesting and wireless power transmission by a hybridized electromagnetic-triboelectric nanogenerator. Energy Environ. Sci. **12**(9), 2678-2684 (2019). https://doi.org/10.1039/c9ee01245a

[S18] Y.C. Wu, X. Wang, Y. Yang, Z.L. Wang, Hybrid energy cell for harvesting mechanical energy from one motion using two approaches. Nano Energy **11**, 162-170 (2015). https://doi.org/10.1016/j.nanoen.2014.10.035

[S19] T. Li, Y. Xu, M. Willander, F. Xing, X. Cao et al., Lightweight triboelectric nanogenerator for energy harvesting and sensing tiny mechanical motion. Adv. Funct. Mater. **26**(24), 4370-4376 (2016). https://doi.org/10.1002/adfm.201600279

[S20] Z.G. Chen, B. Wei, X.M. Mo, C.T. Lim, S. Ramakrishna et al., Mechanical properties of electrospun collagen-chitosan complex single fibers and membrane. Mat. Sci. Eng. C Mater. **29**(8), 2428-2435 (2009). https://doi.org/10.1016/j.msec.2009.07.006

[S21] C.G. Sun, Y. Boluk, C. Ayranci, Investigation of nanofiber nonwoven meshes produced by electrospinning of cellulose nanocrystal suspensions in cellulose acetate solutions. Cellulose **22**(4), 2457-2470 (2015). https://doi.org/10.1007/s10570-015-0665-4

[S22] M.B. Bazbouz, G.K. Stylios, The tensile properties of electrospun nylon 6 single nanofibers. J. Polym. Sci. Polym. Phys. **48**(15), 1719-1731 (2010). https://doi.org/10.1002/polb.21993

[S23] C. Carrizales, S. Pelfrey, R. Rincon, T.M. Eubanks, A.X. Kuang et al., Thermal and mechanical properties of electrospun PMMA, PVC, nylon 6, and nylon 6,6. Polym. Adv. Technol. **19**(2), 124-130 (2008). https://doi.org/10.1002/pat.981

[S24] B. Veleirinho, M.F. Rei, J.A. Lopes-da-Silva, Solvent and concentration effects on the properties of electrospun poly(ethylene terephthalate) nanofiber mats. J. Polym. Sci. Polym. Phys. **46**(5), 460-471 (2008). https://doi.org/10.1002/polb.21380

[S25] F. Croisier, A.S. Duwez, C. Jerome, A.F. Leonard, K.O.V. Werf et al., mechanical testing of electrospun PCL fibers. Acta Biomater. **8**(1), 218-224 (2012). https://doi.org/10.1016/j.actbio.2011.08.015

[S26] C. Liu, J. Shen, K.W.K. Yeung, S.C. Tjong, Development and antibacterial performance of novel polylactic acid-graphene oxide-silver nanoparticle hybrid nanocomposite mats prepared by electrospinning. ACS Biomater. Sci. Eng. **3**(3), 471-486 (2017). https://doi.org/10.1021/acsbiomaterials.6b00766

[S27] A. Abdal-Hay, M. Bartnikowski, S. Hamlet, S. Ivanovski, Electrospun biphasic tubular scaffold with enhanced mechanical properties for vascular tissue engineering. Mat. Sci.

Eng. C Mater. **82**, 10-18 (2018). https://doi.org/10.1016/j.msec.2017.08.041

[S28] J.S. Jeong, J.S. Moon, S.Y. Jeon, J.H. Park, P.S. Alegaonkar et al., Mechanical properties of electrospun PVA/MWNTs composite nanofibers. Thin Solid Films **515**(12), 5136-5141 (2007). https://doi.org/10.1016/j.tsf.2006.10.058

[S29] C. Liu, J. Shen, C.Z. Liao, K.W.K. Yeung, S.C. Tjong, Novel electrospun polyvinylidene fluoride-graphene oxide-silver nanocomposite membranes with protein and bacterial antifouling characteristics. Express Polym. Lett. **12**(4), 365-382 (2018). https://doi.org/10.3144/expresspolymlett.2018.31

[S30] N. Kimura, T. Sakumoto, Y. Mori, K. Wei, B.S. Kim et al., Fabrication and characterization of reinforced electrospun poly(vinylidene fluoride-co-hexafluoropropylene) nanofiber membranes. Compos. Sci. Technol. **92**, 120-125 (2014). https://doi.org/10.1016/j.compscitech.2013.12.002

[S31] Y.K. Liu, W.L. Liu, Z. Wang, W.C. He, Q. Tang et al., Quantifying contact status and the air-breakdown model of charge-excitation triboelectric nanogenerators to maximize charge density. Nat. Commun. **11**, 1599 (2020). https://doi.org/10.1038/s41467-020-15368-9

[S32] W.L. Liu, Z. Wang, G. Wang, G.L. Liu, J. Chen et al., Integrated charge excitation triboelectric nanogenerator. Nat. Commun. **10**, 1426 (2019). https://doi.org/10.1038/s41467-019-09464-8

[S33] L. Cheng, Q. Xu, Y.B. Zheng, X.F. Jia, Y. Qin, A self-improving triboelectric nanogenerator with improved charge density and increased charge accumulation speed. Nat. Commun. 9, 3773 (2018). https://doi.org/10.1038/s41467-018-06045-z

[S34] H.M. Wang, L. Xu, Y. Bai, Z.L. Wang, Pumping up the charge density of a triboelectric nanogenerator by charge-shuttling. Nat. Commun. **11**, 4203 (2020). https://doi.org/10.1038/s41467-020-17891-1

[S35] W. He, W. Liu, J. Chen, Z. Wang, Y. Liu et al., Boosting output performance of sliding mode triboelectric nanogenerator by charge space-accumulation effect. Nat. Commun. 11, 4277 (2020). https://doi.org/10.1038/s41467-020-18086-4

[S36] J.L. Wang, X. Yu, D. Zhao, Y. Yu, Q. Gao et al., Enhancing output performance of triboelectric nanogenerator via charge clamping. Adv. Energy Mater. **11**(31), 2101356 (2021). https://doi.org/10.1002/aenm.202101356
